# Paternal Risk Factors for Oral Clefts in Northern Africans, Southeast Asians, and Central Americans

**DOI:** 10.3390/ijerph14060657

**Published:** 2017-06-19

**Authors:** Stephanie Ly, Madeleine L. Burg, Ugonna Ihenacho, Frederick Brindopke, Allyn Auslander, Kathleen S. Magee, Pedro A. Sanchez-Lara, Thi-Hai-Duc Nguyen, Viet Nguyen, Maria Irene Tangco, Angela Rose Hernandez, Melissa Giron, Fouzia J. Mahmoudi, Yves A. DeClerck, William P. Magee, Jane C. Figueiredo

**Affiliations:** 1 Division of Plastic & Maxillofacial Surgery, Children’s Hospital Los Angeles, Los Angeles, CA 90027, USA; stephaniely@ucla.edu (S.L.); brindopk@usc.edu (F.B.); allyn.auslander@gmail.com (A.A.); WMagee@chla.usc.edu (W.P.M.); 2 Department of Community Health Sciences and California Center for Population Research (CCPR), UCLA Fielding School of Public Health, Los Angeles, CA 90095, USA; 3 Department of Medicine, Keck School of Medicine, University of Southern California, Los Angeles, CA 90033, USA; mburg@usc.edu; 4 Department of Preventive Medicine, Keck School of Medicine, University of Southern California, Los Angeles, CA 90033, USA; Ugonna.Ihenacho@med.usc.edu; 5 Operation Smile Inc., Virginia Beach, VA 23453, USA; kmagee@operationsmile.org; 6 Departments of Pediatrics and Pathology and Laboratory Medicine, Keck School of Medicine, University of Southern California, Children’s Hospital Los Angeles, Los Angeles, CA 90027, USA; PASanchez@chla.usc.edu; 7 Operation Smile Vietnam, Hanoi 100803, Vietnam; thihaiduc.nguyen@operationsmile.org (T.-H.-D.N.); nguyen@operationsmile.org (V.N.); 8 Operation Smile Philippines, Makati City 1209, Philippines; mariatangco@gmail.com (M.I.T.); jello.hernandez@gmail.com (A.R.H.); 9 Department of Surgery, Faculty of Medicine and Surgery, University of Santo Tomas, Manila 1008, Philippines; 10 Operación Sonrisa Honduras, Tegucigalpa 11101, Honduras; Melissa.Giron@operationsmile.org; 11 Operation Smile Morocco, Casablanca 20 000, Morocco; fmahmoudi@operationsmile.org; 12 Departments of Pediatrics and Biochemistry and Molecular Biology, Keck School of Medicine, University of Southern California and Children’s Hospital Los Angeles, Los Angeles, CA 90027, USA; ydeclerck@chla.usc.edu; 13 Department of Medicine, Samuel Oschin Comprehensive Cancer Institute, Cedars-Sinai Medical Center 8700 Beverly Blvd, Room AC1072, Los Angeles, CA 90048, USA

**Keywords:** oral clefts, risk factors, paternal, family history, smoking

## Abstract

While several studies have investigated maternal exposures as risk factors for oral clefts, few have examined paternal factors. We conducted an international multi-centered case–control study to better understand paternal risk exposures for oral clefts (cases = 392 and controls = 234). Participants were recruited from local hospitals and oral cleft repair surgical missions in Vietnam, the Philippines, Honduras, and Morocco. Questionnaires were administered to fathers and mothers separately to elicit risk factor and family history data. Associations between paternal exposures and risk of clefts were assessed using logistic regression adjusting for potential confounders. A father’s personal/family history of clefts was associated with significantly increased risk (adjusted OR: 4.77; 95% CI: 2.41–9.45). No other significant associations were identified for other suspected risk factors, including education (none/primary school v. university adjusted OR: 1.29; 95% CI: 0.74–2.24), advanced paternal age (5-year adjusted OR: 0.98; 95% CI: 0.84–1.16), or pre-pregnancy tobacco use (adjusted OR: 0.96; 95% CI: 0.67–1.37). Although sample size was limited, significantly decreased risks were observed for fathers with selected occupations. Further research is needed to investigate paternal environmental exposures as cleft risk factors.

## 1. Introduction

Oral clefts represent the most common craniofacial birth defect affecting 1–2 per 1000 live births globally with substantial variability by geographic region and ethnic/racial origin, highlighting the importance of genetic and environmental factors [1,2,3]. Research on maternal risk factors from our study [4] and others [5,6,7,8,9] have demonstrated that selected exposures, including nutrition, smoking, alcohol, medications, and chemicals, are associated with oral clefts. Noticeably lacking are data on paternal-specific factors that may influence the risk of oral clefts.

Overall, few paternal factors have been hypothesized to be associated with birth defects, most notably advanced age [10], family history [4,11,12], socioeconomic status (SES) [4,13,14,15,16], smoking [17,18,19,20], and occupational status/exposures [8,21,22]. Advanced paternal age has been shown to be a likely risk factor for oral clefts, although there is significant variability in the definition of “advanced age” across studies [13,23,24,25]. A large meta-analysis found an increased risk of 58% for isolated cleft palate in fathers greater than 40 years old (OR: 1.58, 95% CI: 1.15–2.17) [26]. Paternal family history of clefts has also been consistently identified as a risk factor [4,11,12]. In our prior study using maternal reports of a father’s cleft status, we found an increased risk of oral clefts in children with either fathers or paternal relatives who also had clefts (OR: 10.5, 95% CI: 5.9–18.8) [4]. Paternal smoking has been associated with an increased risk of oral clefts in some studies [4,15,27,28], but not others [5,8,29]. Fathers with lower education levels and/or lower socioeconomic status have been shown to have higher risk of children with oral clefts in several studies [4,13,14,15,16]. Lastly, certain occupational exposures, such as pesticides [6,30,31] have been hypothesized to be associated with a higher risk of clefts with inconclusive evidence. Overall, studies examining paternal exposures have been based on small sample sizes and proxy reports from mothers, and lack specificity to identify specific exposures associated with specific congenital conditions [16,21,32].

In this report, we present an international case–control study on paternal exposures in diverse, underserved populations to further examine the paternal risk factors for clefts independent of maternal factors.

## 2. Methods

### 2.1. Study Population

Methods have been previously published [4]. In brief, we used a case–control study design and collected data on children with cleft lip with or without cleft palate (CL ± P) and cleft palate only (CP) (cases), children without any oral clefts (controls), and their parents in Vietnam, the Philippines, Honduras, and Morocco. Male and female children with a diagnosis of an isolated non-syndromic oral cleft who were singleton births, under age 3, and accompanied by a parent (age ≥18 years) were eligible for inclusion. A pediatrician or clinical geneticist assisted in screening children with oral clefts for other congenital malformations or suspected genetic syndromes, although it was not possible to screen for subclinical or asymptomatic anomalies. Case children were recruited during surgical missions between 2011 and 2015 with Operation Smile, an international not-for-profit organization that specializes in treatment of patients with cleft lip and/or cleft palate, providing millions of patient evaluations and hundreds of thousands of free surgeries across the globe for children and young adults born with craniofacial deformities.

Eligible controls were healthy children of age 3 and under without an oral cleft or other physical congenital malformation at public hospitals in each country from 2011 to 2015. Other exclusion criteria included children who were twins or triplets, children with other congenital anomalies (including limb, craniofacial or skeletal abnormalities), and those whose mothers were pregnant at the time of data collection or had a subsequent pregnancy to reduce the possibility of misreporting exposures unrelated to the pregnancy of interest. Table 1 outlines the dates and location of the data collection sites.

All subjects provided written and/or verbal informed consent. This study was approved by the Institutional Review Board of the University of Southern California (FWA #: 00005906) and the University of Santo Tomas IRB in Manila, Philippines (FWA #: A00009240). In Vietnam, Honduras, and Morocco, collaborating hospital directors reviewed the study and made revisions regarding ethical, cultural, clinical, and vocabulary appropriateness and provided an authorization for human subject research, which were reviewed by the Institutional Review Board of the University of Southern California.

### 2.2. Data Collection and Variable Definitions

Data collection procedures for mothers have been previously published [4]. In this report, we include data collection procedures for interviewing fathers. In brief, all fathers of case and control children were interviewed by a local research assistant at the participating hospitals and clinics in each of the four countries. Research recruiters were trained to interview participants in a standardized manner using a risk factor questionnaire. Fathers and mothers were interviewed in separate areas whenever possible to ensure independent responses. Fathers were asked to report their lifestyle during the periconceptional period (defined as 12–18 months prior to the birth of the child) on demographics, location of residence, alcohol use, tobacco use, health conditions and family history, environmental exposures (i.e., chemicals, radiation), and occupation.

Smoking was defined as regular use of tobacco products, including cigarettes, cigars, and pipes. Alcohol use was defined as regular consumption of wine, beer, or liquor. If present, mothers of these children were also asked to report independently on selected paternal factors, including family history of oral clefting and other health conditions, selected exposures (i.e., smoking), and lifestyle (i.e., educational level, income, and employment status).

### 2.3. Statistical Analysis

To assess potential differences based on parental involvement (mother and father, mother only, or father only), we compared selected characteristics of the study population using chi-square tests for categorical variables (or Fisher’s exact test for variables with small numbers) and Student’s *t*-tests for continuous variables. Data for parental groups with both a participating mother and father (*n* = 626) were used for further analysis in order to adjust for maternal risk factors as reported on the mother’s questionnaire. Logistic regression was used to calculate odds ratios (OR) and 95% confidence intervals (CI) to measure the association between selected self-reported paternal exposures and risk of oral cleft in their children. Adjusted models included the following potential confounders and maternal risk factors: child’s sex, the mother’s place of residence during pregnancy (rural/city), the mother’s and father’s employment status (employed/unemployed), the mother’s and father’s education (completed primary school or less/completed secondary school or more), the mother’s and father’s age at time of delivery, and country. For mothers and fathers missing age at time of delivery, a mean value for age by country and case status was imputed. A “missing” response was created for categorical variables. *p*-values less than 0.05 were considered statistically significant.

Additionally, maternal reports on paternal exposures were compared to results of self-reported paternal data. Kappa statistics were evaluated to assess agreement between mother and father reports. Assessment of adequate agreement was evaluated at the threshold of kappa coefficients greater than 0.70. Assessment of poor agreement was evaluated at threshold of kappa coefficients less than 0.40.

All statistical analysis was performed using SAS software, Version 9.4 of the SAS System for Windows (SAS Institute Inc., Cary, NC, USA).

## 3. Results

Characteristics of the entire study population and the subset with information from fathers are shown in Table 2. There were 626 (24.2%) families with a mother and father that completed a questionnaire, 1895 (73.1%) families with only a mother that completed a questionnaire, and 71 (2.7%) families with only a father that completed a questionnaire. By case status, families with an affected child were more likely to have both parents or only fathers complete a questionnaire compared to control families (*p* < 0.01). There was also a significant difference in the participation of mothers and fathers by country (*p* < 0.01). The majority of families with a participating father and mother, or a mother only, were from Vietnam (40.1% and 36.8%, respectively). The majority of the families with only a participating father were from Morocco (39.4%). More mothers and mothers’ families with clefts were reported among families with a participating mother and father (15.8%, *p* < 0.01), but otherwise there were no significant differences by participant group in maternal factors according to the mother. In regard to paternal factors according to the father, pre-pregnancy alcohol use, tobacco use, and cigarette use was reported more frequently among fathers with participating mothers compared to families with fathers only (*p* < 0.01, *p* = 0.49, and *p* = 0.02, respectively).

Comparing parental reports of paternal factors, we found reasonable agreement among a father’s cleft status (κ: 0.83; 95% CI: 0.67–0.99), a father’s family history of clefts (κ: 0.80; 95% CI: 0.72–0.87), and education (κ: 0.77; 95% CI: 0.72–0.81, Table 3). Moderate agreement was observed for several variables, notably, employment status (κ: 0.68; 95% CI: 0.57–0.79), household tobacco use (κ: 0.65; 95% CI: 0.59–0.71), cigarette use (κ: 0.66; 95% CI: 0.60–0.72), and other tobacco use (κ: 0.66; 95% CI: 0.23–1.00). Poor agreement was found for sexually transmitted infections (HIV and syphilis), diabetes and selected industrial/chemical exposures. Fully concordant responses were given for the assessment of Japanese encephalitis among Vietnamese and Filipino populations and cigar use among the Moroccan mission population (data not shown).

Table 4 provides the measures of association for selected paternal factors and risk of a child with a cleft. Both father’s cleft status (adjusted OR: 3.31; 95% CI: 0.40–27.5) and father’s family history of clefts (adjusted OR: 5.01; 95% CI: 2.46–10.2) were statistically significantly associated with an increased risk (data not shown). When these variables were combined (father’s cleft status and father’s family history of clefts), the adjusted OR was 4.77 (95% CI: 2.41–9.45). Advanced paternal age (5-year adjusted OR: 0.98; 95% CI: 0.84–1.16), pre-pregnancy tobacco use (adjusted OR: 0.96; 95% CI: 0.67–1.37), and pre-pregnancy cigarette use (adjusted OR: 0.95; 95% CI: 0.66–1.37) were not significantly associated with cleft risk. Education level, employment status, and alcohol use were also not statistically significantly associated with risk. When we subdivided occupation into specified categories, exposure to industrial chemicals was associated with a decreased risk of oral cleft (adjusted OR: 0.51; 95% CI: 0.30–0.85) as well as some occupations (mechanics, adjusted OR: 0.54; 95% CI: 0.30–0.95; chemical factory workers, adjusted OR: 0.42; 95% CI: 0.20–0.87; carpenters, adjusted OR: 0.40; 95% CI: 0.23–0.71; and electricians, adjusted OR: 0.47; 95% CI: 0.24–0.94), although sample sizes were limited.

## 4. Discussion

While several maternal exposures have been demonstrated to affect fetal development, paternal exposures are largely unexplored risk factors. Additionally, many studies that report “paternal” risk factors are based on proxy reports from mothers. Here we present data from four international centers in underserved areas to further explore paternal-specific effects on the risk of oral clefts, while accounting for known maternal risk factors. Our results show that a father’s cleft status and family history of clefts are risk factors independent of maternal exposures; however, we found no evidence of paternal environmental risk factors.

The inheritance pattern of oral clefts is thought to be multifactorial, and research has shown a higher recurrence rate in those of affected family members [33], including paternal family history [34]. Our results showed the strongest paternal risk factors to be a father’s cleft status (adjusted OR: 3.31; 95% CI: 0.40–27.5) and a father’s family history of clefts (adjusted OR: 5.01; 95% CI: 2.46–10.2), which supports the important role of genetics in the incidence of oral clefts. Further research is warranted exploring the numbers of generations between affected paternal family members and the child, as well as large population-based studies examining rates of subclinical cleft lip and/or cleft palate amongst fathers of affected children, to fully determine the role of paternal genetics in oral clefts.

Selected paternal occupational and/or chemical exposures have been implicated with a higher incidence of birth defects among their children; however, research specifically examining oral clefts has been inconsistent [8,21,27,35]. The hypothesized mechanism is that occupational exposures may affect DNA integrity prior to conception [36,37]. However, our research found a decreased risk of oral clefts with industrial chemical exposure, along with most fathers who held any type of employment. Although unexpected, these findings among employed fathers may reflect a better standard of living and thus access to prenatal care, due to a higher socioeconomic status, which are associated with lower risk. Further research thoroughly examining the amount and frequency of chemical exposure is needed to confirm this observation.

Previous studies have observed an association between paternal smoking and birth defects among their children [7,15,27]. Our study did not find paternal smoking to be a risk factor after adjustment for other confounders using reports from fathers, in contrast to our previous analysis using proxy reports from mothers (OR: 1.5, 95% CI: 1.1–1.9) [4]. This may reflect the difference in sample size as current reports among father is based on only half the sample size of our prior study focused on mothers. Also of note, data reported from mothers showed a higher proportion of smoking compared to those obtained directly from fathers, which may represent underreporting of tobacco use by fathers. Further research is needed to clarify the role of paternal smoking in oral clefts.

There are several noted strengths and limitations of our study. The data presented here represents one of the largest and most diverse studies focused on paternal-specific exposures. The limitations of this study include a clinic-based study design, biases associated with paternal self-reported data, and a limited sample size. Other factors beyond the scope of our questionnaire that may affect the overall health of the father include atmospheric exposures and water and air quality.

## 5. Conclusions

Oral clefts represent one of the most common birth defects among children born worldwide. Although maternal factors have been studied for decades, there is an important need for studies examining paternal-specific risk factors. Our results suggest that paternal cleft status and family history of clefts are risk factors independent of maternal exposure. We found no evidence of increased risk of oral clefts with paternal environmental risk factors.

## Figures and Tables

**Table 1:** Dates and location of data collection sites.

Country	Cities/Provinces	Dates Collected	Collection Sites
Vietnam	Hanoi, Can Tho, Hai Phong, Hue, Nghe An, Ho Chi Minh City, An Giang	November 2011, November 2012, January 2013, November 2014, March 2015, October–December 2015	Vietnam Cuba Friendship Hospital, Hanoi Maternity Hospital, Hanoi 108 Hospital, Hai Phong Provincial Hospital, Hue Hospital for Odonto-Stomatology, An Giang Hospital, Nghe An 115 Hospital, Thu Duc Hospital, Nghe An Provincial Hospital, Operation Smile Vietnam Care Center, Benh Vien Da Khoa Trung Uong Can Tho Hospital, HCMC Medical Center, Hue Medical and Pharmacy Hospital
Philippines	Bacolod City	November 2012	University of Santo Tomas, HOPE Foundation Cleft Center, Corazon Montelibano Memorial Regional Hospital, Teresita L. Jalandoni Provincial Hospital, Ricardo P. Rodriguez Memorial Hospital, Escolastica Romero District Hospital, Diosdado P. Macapagal Memorial Hospital, Eastern Samar Provincial Hospital, Lying-In Clinic of Borongan City, General Emilio Aguinaldo Memorial Hospital, Paanakan sa Mandaue, Consolacion Municipal Health Office, Grengia Maternity House, St. Anthony Mother and Child Hospital, Our Lady of Mercy Hospital, Jesus A. Datu Medical Center, Operation Smile Care Center: Santa Ana, Miller Adventist Hospital
	Silay City, Angeles City, Manila, Borongan, Dasmarinas City, Cebu, Davao City	June 2014	
	Cebu City, Bacolod City, Pampanga, Santa Ana	June 2015	
Morocco	Marrakesh and Oujda	April and August 2014	Operation Smile Morocco, El Farabi Hospital
	Dahkla and Tiznit	April and August 2015	Operation Smile Morocco, Hospital Hasan II (Dahkla), Hasan I (Tiznit) Hospital
Honduras	Tegucigalpa, Comayagua, Choluteca, Santa Rosa Copan	October 2013, February 2014, June 2014, August 2014, November 2014, February 2015, April 2015, November 2015	San Felipe Hospital, Operation Smile Honduras Clinic, Santa Teresa Regional Hospital, Hospital del Sur, Western Regional Hospital (Hospital Occidente)

**Table 2:** Comparison of families in the study with a participating father and those without a father available to participate.

Characteristics	Families with a Participating Mother and Father	Families with a Non-Participating Father	Families with Non-Participating Mother	*p*-Value *
*N* (%) ^†^	626 (24.2)	1895 (73.1)	71 (2.7)	
Case status				
Case	392 (62.6)	869 (45.9)	45 (63.4)	<0.01 **
Control	234 (37.4)	1021 (53.9)	25 (35.2)	
Missing	0 (0)	5 (0.3)	1 (1.4)	
Country				
Vietnam	251 (40.1)	697 (36.8)	14 (19.7)	<0.01
Philippines	195 (31.2)	530 (28.0)	18 (25.4)	
Honduras	119 (19.0)	546 (28.8)	11 (15.5)	
Morocco	61 (9.74)	122 (6.4)	28 (39.4)	
Sex				
Male	384 (61.3)	1051 (55.5)	N/A	<0.01 **
Female	239 (38.2)	841 (44.4)		
Missing	3 (0.5)	3 (0.2)		
Cleft type				
CL	99 (25.3)	225 (25.9)	N/A	0.98
CLP	220 (56.1)	490 (56.4)		
CP	67 (17.1)	140 (16.1)		
Missing	6 (1.5)	14 (1.6)		
MATERNAL FACTORS—according to mother				
Age at birth, years				
mean (SD)	27.2 (6.0)	27.2 (6.1)	Not collected	0.89
Education level				
University	159 (25.4)	461 (24.3)	Not collected	0.26
Secondary	313 (50.0)	934 (49.3)		
None/Primary	150 (24.0)	468 (24.7)		
Missing	4 (0.6)	32 (1.7)		
Employment status				
Unemployed	340 (54.3)	1040 (54.9)	Not collected	0.91
Employed	275 (43.9)	818 (43.2)		
Missing	11 (1.8)	37 (2.0)		
Mother cleft status & mother’s family history of clefts				
Yes	99 (15.8)	211 (11.1)	Not collected	<0.01
No	525 (83.9)	1675 (88.4)		
Missing	2 (0.3)	9 (0.5)		
Location during pregnancy				
Rural	277 (44.3)	820 (43.3)	Not collected	0.76
City	301 (48.1)	913 (48.2)		
Missing	48 (7.7)	162 (8.6)		
PATERNAL FACTORS—according to father				
Age at birth, years				
mean (SD)	30.6 (7.0)	Not collected	31.6 (7.5)	0.26
Education level				
University	178 (28.4)	Not collected	18 (25.4)	0.33
Secondary	296 (47.3)		29 (40.9)	
None/Primary	141 (22.5)		23 (32.4)	
Missing	11 (1.8)		1 (1.4)	
Pre-pregnancy employment status				
Unemployed	26 (4.2)	Not collected	2 (2.8)	0.93 **
Employed	579 (92.5)		67 (94.4)	
Missing	21 (3.4)		2 (2.8)	
Father’s cleft status & father’s family history of clefts				
Yes	83 (13.3)	Not collected	13 (18.3)	0.23
No	543 (86.7)		58 (31.7)	
Missing	0 (0)		0 (0)	
Chemical exposures				
Industrial chemical	79 (12.6)	Not collected	6 (8.5)	0.31
Agricultural chemical	188 (30.0)		17 (23.9)	0.29
Radiation	41 (6.6)		5 (7.0)	0.80 **
Lead	21 (3.5)		1 (1.4)	0.50 **
Mercury	11 (1.8)		0 (0)	0.61 **
Chemical waste	24 (3.8)		0 (0)	0.16 **
Agent Orange (Vietnam only)	6 (2.4)		0(0)	0.56
Pre-pregnancy alcohol use				
Yes	409 (65.3)	Not collected	27 (38.0)	<0.01 **
No	213 (34.0)		43 (60.6)	
Missing	4 (0.6)		1 (1.4)	
Pre-pregnancy tobacco use				
Yes	244 (39.0)	Not collected	18 (25.4)	0.049
No	376 (60.1)		53 (74.7)	
Missing	6 (1.0)		0 (0)	
Frequency of tobacco use				
<1	22 (9.0)	Not collected	2 (11.1)	0.90 **
1–3	47 (19.3)		4 (22.2)	
6–14	47 (19.3)		5 (27.8)	
15–20	41 (16.8)		2 (11.1)	
20+	82 (33.6)		5 (27.8)	
Missing	5 (2.1)		0 (0)	
Pre-pregnancy cigarette use				
Yes	232 (37.1)	Not collected	16 (22.5)	0.02
No	384 (61.3)		55 (77.5)	
Missing	10 (1.6)		0 (0)	
Frequency of cigarette use				
<1	22 (9.5)	Not collected	2 (12.5)	0.82 **
1–3	42 (18.1)		4 (25.0)	
6–14	44 (19.0)		4 (25.0)	
15–20	39 (16.8)		1 (6.3)	
20+	78 (33.6)		5 (31.3)	
Missing	7 (3.0)		0 (0)	
Father’s occupation				
Farmer	272 (43.5)	Not collected	26 (36.6)	0.27
Driver	104 (16.6)		11 (15.5)	0.81
Mechanic	60 (9.6)		4 (5.6)	0.27
Factory worker	35 (5.6)		6 (8.5)	0.29 **
Carpenter	62 (9.9)		11 (15.5)	0.15
Painter	65 (10.4)		3 (4.2)	0.10
Welder	58 (9.3)		6 (8.5)	0.82
Electrician	41 (6.6)		3 (4.2)	0.61 **

* *X*^2^ or ANOVA to for test for differences between families with and without a participating father. ^†^ Data given as number and column percentages unless otherwise noted. ** *p*-value given by Fisher’s Exact Test.

**Table 3:** Kappa statistics on paternal exposures based on questionnaires completed by mothers and fathers *.

Variable	*N* **	Kappa (95% CI)	*p*-Value
Father’s cleft status	598	0.83 (0.67–0.99)	<0.0001
Father’s family history of clefts	600	0.80 (0.72–0.87)	<0.0001
Education	597	0.77 (0.72–0.81)	<0.0001
Employment status	597	0.68 (0.57–0.79)	<0.0001
Malaria	532	0.70 (0.54–0.85)	<0.0001
Typhoid	580	0.51 (0.26–0.76)	<0.0001
Hepatitis	583	0.62 (0.44–0.80)	<0.0001
Dengue	582	0.65 (0.54–0.77)	<0.0001
Meningitis	538	0 (0–0)	-
HIV	582	−0.002 (−0.004–0.001)	0.97
Syphilis	531	−0.002 (−0.005–0.001)	0.97
Birth defect	585	0.50 (−0.10–1.00)	<0.0001
Vision defect	586	0.69 (0.52–0.87)	<0.0001
Hearing defect	585	0.44 (0.03–0.85)	<0.0001
Diabetes	579	−0.002 (−0.006–0.001)	0.95
Other condition	483	0.47 (0.22–0.71)	<0.0001
Household tobacco use	608	0.65 (0.59–0.71)	<0.0001
Household tobacco use frequency ^‡^ (*N* = 215)	206	0.59 (0.50–0.68)	<0.0001
Cigarette use	595	0.66 (0.60–0.72)	<0.0001
Other tobacco use	371	0.66 (0.23–1.00)	<0.0001
Industrial chemical exposure	547	0.38 (0.27–0.50)	<0.0001
Agricultural chemicals exposure	546	0.51 (0.43–0.59)	<0.0001
Thuoc lao use—Vietnam only (*N* = 251)	112	0.76 (0.61–0.92)	<0.0001
Agent orange exposure—Vietnam only (*N* = 251)	225	0.24 (−0.16–0.64)	<0.0001
Tuberculosis—Vietnam only (*N* = 251)	234	0.80 (0.52–1.00)	<0.0001

* Evaluated for questions where both mother and father provided a response (missing responses excluded from analysis). ** Total *N* varies due to missing values. ^‡^ Weighted kappa reported.

**Table 4:** Paternal health status and exposures prior to conception and the risk of a child with an orofacial cleft.

	Cases	Controls	Crude OR (95% CI)	Adjusted OR (95% CI)
*N* (%)	392 (62.6)	234 (37.4)		
* Age at birth, years				
mean (SD)	31.0 (7.3)	30.0 (6.4)	1.11 (0.99–1.25)	0.98 (0.84–1.16)
Father’s and father’s family history of clefts				
Yes	72 (18.4)	11 (4.7)	4.66 (2.40–9.01) ^†^	4.77 (2.41–9.45) ^†^
No	320 (81.6)	223 (95.3)	Ref	Ref
Education level				
University	93 (23.7)	85 (36.3)	Ref	Ref
Secondary	195 (49.7)	101 (43.2)	1.77 (1.21–2.59) ^†^	1.47 (0.97–2.21)
None/Primary	94 (24.0)	47 (20.1)	1.89 (1.17–3.06) ^†^	1.29 (0.74–2.24)
Missing	10 (2.6)	1 (0.4)		
Pre-pregnancy employment status				
Unemployed	12 (3.1)	14 (6.0)	Ref	Ref
Employed	366 (93.4)	213 (91.0)	2.03 (0.92–4.50)	2.08 (0.90–4.85)
Missing	14 (3.6)	7 (3.0)		
Pre-pregnancy alcohol use				
Yes	247 (63.0)	162 (69.2)	0.70 (0.46–1.06)	0.77 (0.49–1.21)
No	141 (36.0)	72 (30.8)	Ref	Ref
Missing	4 (1.0)	0 (0)		
Pre-pregnancy tobacco use				
Yes	149 (38.0)	95 (40.6)	0.91 (0.65–1.27)	0.96 (0.67–1.37)
No	238 (60.7)	138 (59.0)	Ref	Ref
Missing	5 (1.3)	1 (0.4)		
Frequency of tobacco use				
<1	17 (11.4)	5 (5.3)	Ref	Ref
1–3	28 (18.8)	19 (20.0)	0.39 (0.12–1.26)	0.42 (0.12–1.45)
6–14	28 (18.8)	19 (20.0)	0.37 (0.11–1.20)	0.46 (0.13–1.64)
15–20	27 (18.1)	14 (14.7)	0.52 (0.16–1.72)	0.65 (0.18–2.34)
20+	44 (29.5)	38 (40.0)	0.29 (0.10–0.89) ^†^	0.34 (0.10–1.12)
Missing	5 (3.4)	0 (0)		
Pre-pregnancy cigarette use				
Yes	140 (35.7)	92 (39.3)	0.88 (0.63–1.23)	0.95 (0.66–1.37)
No	243 (62.0)	141 (60.3)	Ref	Ref
Missing	9 (2.3)	1 (0.4)		
Frequency of cigarette use				
<1	17 (12.1)	5 (5.4)	Ref	Ref
1–3	24 (17.1)	18 (19.6)	0.37 (0.11–1.20)	0.40 (0.11–1.42)
6–14	25 (17.9)	19 (20.7)	0.34 (0.10–1.10)	0.41 (0.11–1.49)
15–20	26 (18.6)	13 (14.1)	0.54 (0.16–1.81)	0.68 (0.19–2.52)
20+	42 (30.0)	36 (39.1)	0.31 (0.21–23.6)	0.33 (0.10–1.11)
Missing	6 (4.3)	1 (1.1)		
Chemical exposures				
Industrial chemical	40 (10.2)	39 (16.7)	0.57 (0.35–0.91) ^†^	0.51 (0.30–0.85) ^†^
Agricultural chemical	129 (32.9)	59 (25.2)	1.47 (1.02–2.12) ^†^	0.99 (0.66–1.49)
Radiation	20 (5.1)	21 (9.0)	1.83 (0.97–3.46)	0.66 (0.33–1.33)
Lead	10 (2.6)	12 (5.1)	0.48 (0.21–1.14)	0.39 (0.15–1.01)
Mercury	5 (1.3)	6 (2.6)	0.49 (0.15–1.63)	0.37 (0.09–1.47)
Chemical waste	12 (3.1)	12 (5.1)	0.58 (0.26–1.32)	0.58 (0.24–1.42)
Agent Orange (Vietnam only)	5 (3.0)	1 (1.2)	2.61 (0.30–22.7)	3.06 (0.22–42.5)
Father’s occupation				
Farmer	188 (48.0)	84 (35.9)	1.66 (1.19–2.32) ^†^	1.12 (0.76–1.65)
Driver	64 (16.3)	40 (17.1)	0.95 (0.61–1.46)	1.03 (0.65–1.65)
Mechanic	27 (6.9)	33 (14.1)	0.45 (0.26–0.77) ^†^	0.54 (0.30–0.95) ^†^
Factory worker	15 (3.8)	20 (8.6)	0.43 (0.21–0.85) ^†^	0.42 (0.20–0.87) ^†^
Carpenter	27 (6.9)	35 (15.0)	0.42 (0.25–0.71) ^†^	0.40 (0.23–0.71) ^†^
Painter	37 (9.4)	28 (12.0)	0.76 (0.45–1.29)	0.77 (0.44–1.35)
Welder	33 (8.4)	25 (10.7)	0.77 (0.45–1.33)	0.97 (0.54–1.74)
Electrician	18 (4.6)	23 (9.8)	0.44 (0.23–0.84) ^†^	0.47 (0.24–0.94) ^†^

Adjusted by child’s sex, mother’s place of residence during pregnancy (rural/city), mother’s and father’s employment status (employed/unemployed), mother’s and father’s education (completed primary school or less/completed secondary school or more), mother’s and father’s age at time of delivery and country. * 5 year OR given. Crude models were adjusted by country. ^†^ *p* is significant at the 0.05 level.
